# Diagnostic Challenges in Aortic Stenosis

**DOI:** 10.3390/jcdd11060162

**Published:** 2024-05-23

**Authors:** André González-García, Pablo Pazos-López, Francisco Eugenio Calvo-Iglesias, Tatiana Mallely Matajira-Chía, Raquel Bilbao-Quesada, Elisa Blanco-González, Carina González-Ríos, María Castiñeira-Busto, Manuel Barreiro-Pérez, Andrés Íñiguez-Romo

**Affiliations:** Department of Cardiology, Hospital Alvaro Cunqueiro, Complexo Hospitalario Universitario de Vigo, 36312 Vigo, Spain; francisco.calvo.iglesias@sergas.es (F.E.C.-I.); tatiana.mallely.matajira.chia@sergas.es (T.M.M.-C.); raquel.bilbao.quesada@sergas.es (R.B.-Q.); elisa.blanco.gonzalez@sergas.es (E.B.-G.); carina.gonzalez.rios@sergas.es (C.G.-R.); maria.castineira.busto@sergas.es (M.C.-B.); manuel.barreiro.perez@sergas.es (M.B.-P.); andres.iniguez.romo@sergas.es (A.Í.-R.)

**Keywords:** aortic stenosis, multimodality cardiac imaging, continuity equation, low-flow aortic stenosis, discordant aortic stenosis, planimetry, multidetector computed tomography, cardiac magnetic resonance

## Abstract

Aortic stenosis (AS) is the most prevalent degenerative valvular disease in western countries. Transthoracic echocardiography (TTE) is considered, nowadays, to be the main imaging technique for the work-up of AS due to high availability, safety, low cost, and excellent capacity to evaluate aortic valve (AV) morphology and function. Despite the diagnosis of AS being considered straightforward for a very long time, based on high gradients and reduced aortic valve area (AVA), many patients with AS represent a real dilemma for cardiologist. On the one hand, the acoustic window may be inadequate and the TTE limited in some cases. On the other hand, a growing body of evidence shows that patients with low gradients (due to systolic dysfunction, concentric hypertrophy or coexistence of another valve disease such as mitral stenosis or regurgitation) may develop severe AS (low-flow low-gradient severe AS) with a similar or even worse prognosis. The use of complementary imaging techniques such as transesophageal echocardiography (TEE), multidetector computed tomography (MDTC), or cardiac magnetic resonance (CMR) plays a key role in such scenarios. The aim of this review is to summarize the diagnostic challenges associated with patients with AS and the advantages of a comprehensive multimodality cardiac imaging (MCI) approach to reach a precise grading of the disease, a crucial factor to warrant an adequate management of patients.

## 1. Introduction

Aortic stenosis (AS) is a relevant entity in daily practice due to several factors. It is the most common heart valve disease in developed nations [1], with an increased occurrence in the elderly (10–15% of individuals over 80 years of age) [2,3]. Bearing in mind the population aging of western countries, it is expected that AS prevalence may double by 2050. Another matter of concern is the high morbidity and mortality of this pathology at advanced stages; prognosis of symptomatic severe AS patients without percutaneous or surgical treatment is still ominous nowadays. Diagnosis and classification of AS was considered quite simple in the past, based on transvalvular flow maximal velocity (VMax), mean gradient (MG), and valve area (AVA) which could be obtained with transthoracic echocardiography (TTE). However, several investigations have demonstrated that this is a simplistic approach. Patients at a high output state (due to anemia, veno–arterial shunts) can exhibit a significant rise in gradients in the presence of just moderate AS, whereas a low-flow (LF) state (due to left ventricular systolic dysfunction, concentric left ventricular hypertrophy) may justify a true severe AS with a VMax and MG not particularly elevated. A multimodality cardiac imaging approach (MCI) plays a key role in these scenarios. The addition of complementary techniques such as transesophageal echocardiography (TEE), multidetector computed tomography (MDCT), and cardiac magnetic resonance (CMR) allows a proper grading in most cases.

The objective of this review is to delve into the MCI used in the assessment of AS, highlighting the usefulness, strengths, and weaknesses of each imaging test, particularly in specific cases in which the grading of the disease is challenging.

## 2. Optimal Assessment of Aortic Stenosis by Multimodality Cardiac Imaging

### 2.1. Transthoracic Echocardiography

#### 2.1.1. General Principles of Transthoracic Echocardiography Exam

Two-dimensional (2D) TTE is the first step and corner stone in the evaluation of a suspected AS due to its high availability, safety, low cost, and excellent ability to appraise the morphology and function of the aortic valve (AV). TTE is conclusive on most occasions and no further imaging is needed for clinical decision making. Ideally, the hemodynamic status of the patient at the time of the TTE scanning should be stable and certain parameters such as blood pressure and heart rate well controlled in order to avoid their influence on echo estimations (i.e., a high afterload caused by hypertension or a decreased diastolic phase of the cardiac cycle in the context of rapid atrial fibrillation (AF) may lead to a significant reduction of transaortic valve gradient).

**Figure 1:**
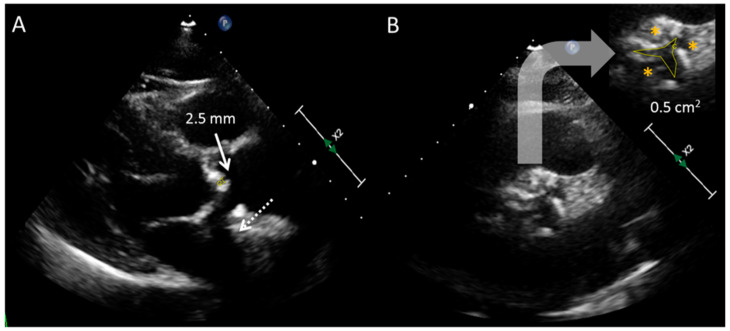
Morphological evaluation of the aortic valve by TTE. (**A**) Parasternal long-axis view. Extensive calcification of the aortic valve leaflets is noted (dashed arrow: acoustic shadow generated by the calcium). Maximal systolic separation between cusps reaches only 2.5 mm, a finding supporting the existence of a significant stenosis (classical cut off for severe AS: <7–8 mm). (**B**) Parasternal short-axis view. Three leaflets (asterisks) are identified; at the systole, the aortic valve orifice is significantly reduced (area by direct tracing or “planimetry” is 0.5 cm^2^).

**Figure 2:**
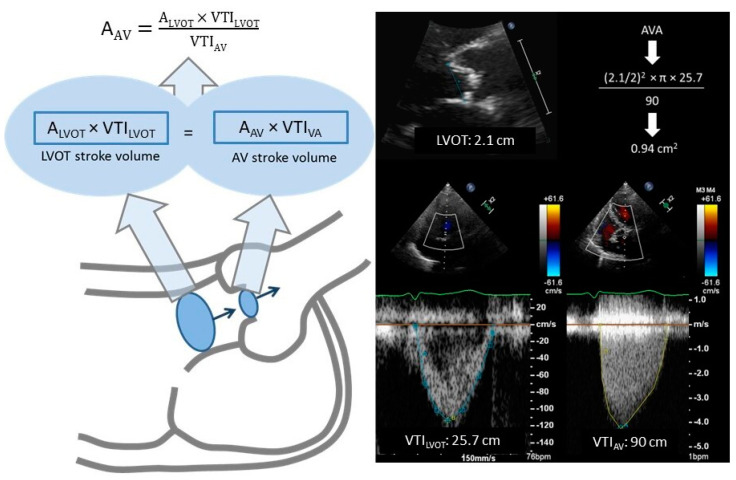
Continuity equation. A_AV_: aortic valve area, A_LVOT_: LVOT area, estimated via the circle area formula: [(Diameter/2)^2^ × π], VTI_AV_: velocity time integral at the aortic valve orifice (calculated with CWD aligning the ultrasound beam parallel to the systolic blood flow through the aortic valve), VTI_LVOT_: velocity time integral at LVOT (assessed with PWD).

Long and short axis views are employed in every TTE examination. Parasternal long axis view (PLAV) helps to assess qualitatively the valve cusps thickening, calcification, and opening [4] (see Figure 1). Color Doppler interrogation determines the occurrence of flow acceleration through the AV (characterized by a “mosaic pattern”), an indirect sign of a restrictive valve aperture, and the coexistence of regurgitation. This view is selected to measure the left ventricular outflow track diameter (LVOT), which is recommended to be done at the level of the annulus in mid-systole [5]. At this time point, opening of the AV is maximal and LVOT acquires its most circular shape and largest area [6]. LVOT size is needed to calculate the AVA through the continuity equation (CEq) (see Figure 2). This component of the formula represents its main source of error [7], so care should be taken (zooming is recommended) to ensure the highest precision in such measurement.

AV leaflets are visualized from the short-axis view (SAV). SAV offers complemental information about valve morphology that leads to the determination of the etiology of the stenosis (i.e., commissural fusion is a typical feature of rheumatic disease; bicuspid valves depict two instead of three cusps). Despite the anatomical orifice area may be measurable by direct tracing (“planimetry”; see Figure 1), technical aspects such as the angulation of the echocardiographic plane or the quality of the echocardiographic window make such an assessment inaccurate in most patients.

Apical 5-chamber (A5C) and 3-chamber views (A3C) are the standard views for a functional evaluation of AV. Pulsed wave Doppler (PWD) and continuous wave Doppler (CWD) interrogations are used in this regard. PWD at the LVOT allows a velocity time integral (VTI) assessment at this site, a component of the CEq. A sample volume should be initially placed at the AV and then moved apically until an optimal spectral curve is obtained. CWD is employed to estimate VMax and MG. Alignment between the ultrasound bean (US) and the flow jet should be parallel to elude underestimation of transvalvular velocity and, consequently, gradient [8].

#### 2.1.2. Transthoracic Echocardiography Parameters Used in Aortic Stenosis Quantification

**Figure 3:**
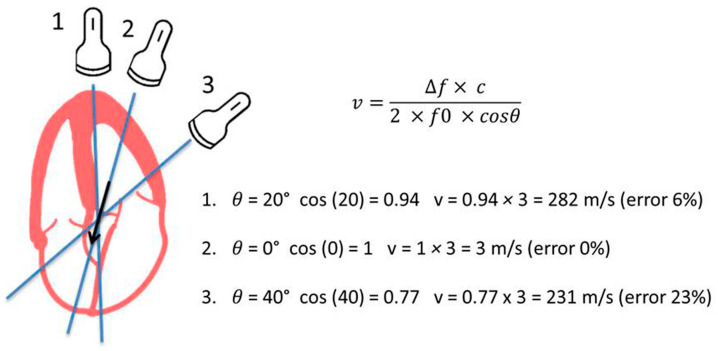
Effect of the US-beam–aortic-valve blood flow angle on the assessment of transvalvular aortic velocity. Using the Doppler formula, blood flow velocity (v) can be estimated by calculating the frequency difference between emitted (*f*0) and reflected US (∆f) and the cosine of the angle between the US beam and the blood flow (*θ*). C refers to the speed of US in blood (1.540 ms). In this example, we set a theoretical real maximum transvalvular aortic velocity of 3 m/s. Three different positions of the US probe (1–3) that allow different alignments between the US beam (blue lines) and the flow (black arrow) are represented. Neglecting *θ* and assuming it to be 0 at all transducer locations will result in calculation errors. In 1 (*θ* = 20°), such an error is 6%, while, in example 3, given the greater obliquity (*θ* = 40°), it is 23%. Echocardiography software set *θ* = 0° by default.

CWD modality, which analyzes the flows found in the path of the US beam, is employed to assess blood velocity through the AV. Gradients are estimated using the simplified Bernoulli equation (4 × velocity^2^). VMax is the parameter with the greatest amount of scientific evidence, based on prospective studies, to predict cardiovascular events in patients with AS [9,10,11]. MG is calculated by plotting the CWD spectral curve; the US machine or workstation software averages the maximum derived gradients at each point of the curve. It has been established that CWD-derived MG correlates closely with the corresponding gradient by catheterization [12]. The main limitations of VMax and MG are as follows:

- Interposition of air or valvular calcium may preclude US penetration, so the identification of the envelope of the Doppler spectral curve may be difficult in some cases.
- Assessment of transvalvular flow velocity by Doppler technique is angle-dependent (see Figure 3).
- The simplified Bernoulli equation can be unsuitable in cases where high LVOT velocities are present (i.e., subaortic membrane or obstructive hypertrophic cardiomyopathy).
- VMax and MG are flow-dependent parameters: high-flow states (i.e., fever, anemia, significant aortic or mitral regurgitation) or low-flow states (i.e., left ventricular systolic dysfunction or significant mitral stenosis) may, therefore, cause inaccuracy in AS grading.
- Pressure recovery. The conversion of potential energy into kinetic energy due to the passage of blood through the VA leads to an increase in flow rate and a drop in pressure. Although some of the kinetic energy is dissipated as heat, due to turbulence and viscous losses, distal to the stenosis at the level of the aortic root, another part is transformed back into potential energy which causes a deceleration of flow and an increase or recovery of pressure (pressure recovery phenomenon; PRF). When the blood flow penetrates the AV, the anatomic orifice pressure keeps falling and velocity increases over a short distance, leading to the formation of what we call vena contracta (VC) which represents the effective orifice of the AV (EOAV). EOAV is slightly smaller than the anatomic orifice area and the major determinant of survival for the patients with AS [5]. VMax and the derived maximal gradient estimated by CWD reflect the flow velocity and pressure drop at the VC, hence PRF is not taken into consideration by Doppler techniques. In most adults with AS, the magnitude of PRF is small when the diameter of the ascending aorta is >30 mm. However, in patients with a smaller aortic caliber, PRF may be significant and, therefore, VMax and gradients may overestimate AS [13,14].

By dint of the CEq, TTE is able to assess AVA (see Figure 2). CEq is based on the principle of mass conservation, which equates stroke volume (SV) proximal to the aortic valve to SV through the stenotic AV orifice [15]. Proximal SV corresponds to LVOT-SV (calculated by multiplying the LVOT area by VTI at this site). VTI is obtained with the PWD spectral curve and LVOT area is indirectly derived from LVOT diameter, with the assumption that LVOT cross-section is a perfect circumference. AVA estimated via CEq has been validated against catheterization-derived AVA as a reference [12,16,17]. In contrast to VMax, publications that have demonstrated an independent association between AVA and survival are scarce. Most data suggest that an AVA <0.8–1 cm^2^ or an indexed AVA (AVAi) <0.5–0.6 cm^2^ carry a poor prognosis [18,19]. Listed below are the main limitations regarding AVA calculation with the CEq.

- Changes in transvalvular flow rate are potential modifiers of AVA. This effect is marginal in the context of preserved left ventricular ejection fraction (LVEF). However, it may be important when LV systolic disfunction is present, as a significant reduction of the AV orifice, in the absence of severe AS, may occur if the LV is unable to generate the required energy to adequately open the AV [20].
- Inter- and intraobserver reproducibility for LVOT diameter measurements is the main source of inaccuracy relative to the CEq. Although variability in such measurements is only ≈5 ± 4% and ≈3 ± 2%, respectively [21], it may be meaningful because this parameter is squared in CEq.
- LVOT real shape is elliptical, whereas a round configuration is considered in the CEq formula. This is the cause of up to 20% underestimation of the AVA by the CEq [22,23].
- LVOT-VTI measurement is based on the acceptance that flow is laminar with a flat spatial distribution at that site. When the LVOT-VTI assessment is performed distal to the AV annulus, flow convergence may generate higher velocities in the region adjacent to the interventricular septum and lower velocities in the proximity of the mitral valve. In this case, a non-centered PWD sample volume location can lead to over or underestimation of the systolic SV at the LVOT, resulting in AVA calculation inaccuracies.

#### 2.1.3. Other Useful TTE Measurements (See Table 1)

**Figure 4:**
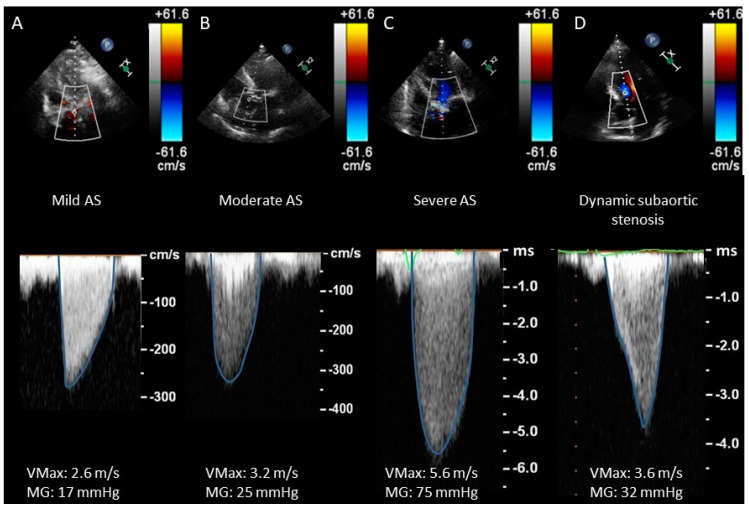
Grading of AS by VMax and MG. (**A**) Mild AS. The morphology of the CWD spectral curve is triangular; an early VMax can be appreciated. As the stenosis progresses ((**B**): moderate AS, (**C**): severe AS), the curve becomes rounded and VMax is delayed. (**D**) Dynamic subvalvular stenosis. In this case, the spectral curve acquires a characteristic “dagger” shape with a late VMax. AS: aortic stenosis. MG: mean gradient. VMax: transvalvular flow maximal velocity.

- AVAi: it is defined as the ratio of AVA to body surface area (BSA). It should be used with caution in overweight patients because it significantly increases the prevalence of severe AS criteria without improving the predictive accuracy for AV-related events [24]. In contrast, AVAi can reclassify AS to a lower degree in a significant proportion of subjects with a small body habitus (body surface area < 1.7 m^2^).
- Dimensionless index (DI): it is calculated from the ratio of LVOT velocity to trans-AV velocity. Despite the potential advantage of avoiding LVOT diameter, investigations have demonstrated that it is less accurate than AVA [16,17]. A cut-off of <0.25 reaches a sensitivity and specificity with respect to AVA <0.75 cm^2^ of 92% and 78%, respectively [25].
- Systemic arterial compliance: it is a novel parameter that has been proposed to evaluate AS severity. Validation is still needed to recommend its use for AS grading [26].
- CWD waveform (see Figure 4) is a semiquantitative parameter to evaluate the severity of AS. The finding of a CW waveform with a rapid acceleration and an early peak makes severe AS very unlikely. On the other hand, a slower acceleration with a late peak is more specific to severe AS. A dagger-shaped pattern may correspond to a dynamic subvalvular aortic stenosis (a common feature of obstructive hypertrophic cardiomyopathy) [27].

**Table 1:** Cut-off values of the different TTE parameters used to grade AS. Source: own elaboration.

	No AS	Mild AS	Moderate AS	Severe AS
VMax (m/s)	<2.5	2.5–3	3–4	≥4
MG (mmHg)	-	<20	20–40	≥40
AVA (cm^2^)	>2	1.5–2	1–1.5	<1
AVA index (cm^2^/m^2^)	-	>0.85	0.6–0.85	<0.6
DI	-	>0.50	0.25–0.50	<0.25

- Three-dimensional (3D) TTE acquisition: a 3D echo dataset allows direct LVOT measurement by multiplanar reformat, in a similar way MDCT does, avoiding geometrical assumptions. LVOT assessment by 3D TTE is more reproducible than 2D TTE and can be employed in the CEq (“ellipsis formula”) [23,28]. Three-dimensional probes are not available in many centers and, despite recent technological advances, the spatial and temporal resolution of 3D TTE is low, so its role may still be limited.
- Chamber’s size, left ventricle wall thickness, systolic and diastolic function, and pulmonary pressure are easily obtained by TTE. All these parameters, as well as the presence of another concomitant valve dysfunction that can be diagnosed within the same exam, are useful for decision making with respect to patients with AS.

### 2.2. Transesophageal Echocardiography

**Figure 5:**
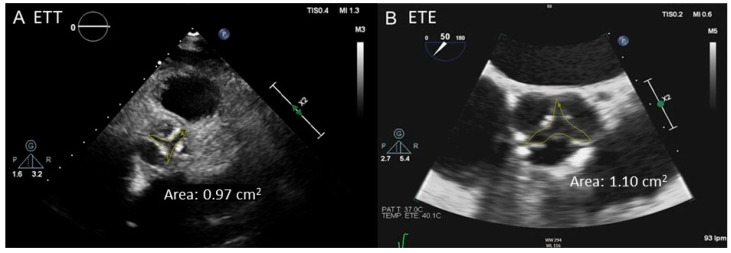
Aortic valve planimetry using TTE (**A**) vs. TEE (**B**). A better definition of the free edges of the leaflets with TEE facilitates the tracing; in this case, the valve orifice area moves from 0.97 cm^2^ ((**A**) severe stenosis) to 1.10 cm^2^ ((**B**) moderate stenosis). TEE: transesophageal echocardiography. TTE: transthoracic echocardiography.

TEE can be helpful for AV morphological evaluation, although grading AS using Doppler techniques is challenging because of the difficulty in aligning the US beam with the valve orifice. Due to the anatomical proximity of the transducer to the AV and absence of pulmonary air interposition, TEE can visualize the valve structure with excellent definition in most patients. The feasibility of aortic AV planimetry is superior to that of TTE (90–95% according to different reports, see Figure 5) [29,30,31]. However, in cases of extensive leaflets calcification, the precision of this measurement may be reduced. Additionally, TEE is a semi-invasive test that requires sedation. For these reasons, TEE is reserved for selected cases (i.e., patients with high suspicion of severe AS and inconclusive TTE study due to poor transthoracic window).

Three-dimensional TEE overcomes the main limitation of its 2D counterpart: the dependence of the scanning angle. Planimetry with this modality has shown better reproducibility than that of the 2D version [32], although it suffers from the same limitations in patients with significant valve calcification [33]. LVOT imaging with 3D TEE can be used to obtain its “real” size and has been demonstrated to reduce the proportion of patients with severe AS by isolated AVA criteria, reclassifying a significant proportion (up to 40% of cases) as moderate AS [34,35] and leading, therefore, to a decrease in AVA gradient discrepancies [35].

### 2.3. Multidetector Computed Tomography

MDCT features a high spatial resolution, which allows an adequate anatomical assessment of the AV but does not provide functional information. The limitations of CT are well-known (exposure to ionizing radiation and the need to use iodinated contrast media, which are particularly harmful in patients with impaired renal function). The main applications of CT can be summarized as follows:

- Measurement valve calcification. The presence and extent of valvular calcification, quantified by the calcium score using the Agatston method, has been shown to be a predictor of severe AS, as well as of disease progression and development of adverse events [36]. The main application of the AV calcium score is the differentiation between true severe AS and pseudosevere AS, especially in the presence of preserved LVEF, LF, and significant diastolic dysfunction, i.e., patients with a restrictive diastolic pattern whose tolerance to dobutamine stress echocardiography (DSE) is limited. Cut-off values for severe AS vary according to gender (≥1200 Agatston Units (AU) in women and ≥2000 AU in men; valvular calcium density (AU/aortic annulus area) ≥300 AU/cm^2^ in women and ≥500 AU/cm^2^ in men [4,14,36,37,38]). In patients with concordant echocardiographic measurements of disease severity, the CT calcium score has a sensitivity and specificity of 87% and 84%, respectively, in females and of 80% and 82%, respectively, in males for identifying severe AS [39]. In contrast, in individuals with discordant TTE parameters, heterogeneity in CT calcium scores has been observed [39]. Severe valve calcification is present in ≈50–60% of the patients with LF AS, whereas, among discordant normal flow (NF) AS, it has been detected in 74% of the subjects with a VMax > 4.0 m/s, but only in 34% of the individuals with a VMax < 4.0 m/s [39]. It should be noted that CT does not assess AV fibrosis, which may contribute significantly to AS in some cases, such as in young individuals with bicuspid AV, although the proportion of such patients in LF scenarios is very low.
- Planimetry. MDCT allows 3D acquisition throughout the entire cardiac cycle using retrospective protocols. Through multislice reconstructions, planimetry of the AV orifice is feasible. Such a measure has shown good agreement with AVA estimated through the CEq [40]. Concerning LVOT size, MDCT assessment is considered to be the gold standard test [23]. Clavel et al. [41] observed that AVA calculated with a hybrid TTE–MDCT method in which the LVOT area used in the Ceq was obtained with MDCT predicted long-term survival with an optimal cut-off value of 1.2 cm^2^ instead of 1 cm^2^ [41].
- Pre-interventional study. Coverage of MDCT at acquisition can be extended to the ascending aorta to measure its diameter and determine the presence of calcifications. This information is helpful in cases where surgical valve replacement is considered. Regarding transcatheter aortic valve replacement (TAVR), CT is the reference technique for procedural planning. MDCT allows a precise estimation of the valve annulus size, its distance to the coronary ostia, and the caliber of the peripheral arterial vascular accesses. All of these parameters are essential to determine the candidacy for TAVR and to select the type and size of the prosthesis. Moreover, the incorporation of coronary assessment into CT protocols does not require an increase in contrast or radiation doses and has been shown to reduce the need for invasive coronary angiography in a noteworthy percentage of cases [42]. The use of the novel CT-derived fractional flow reserve technique is still not recommended because it may increase the number of false positive tests [43].

### 2.4. Cardiac Magnetic Resonance

**Figure 6:**
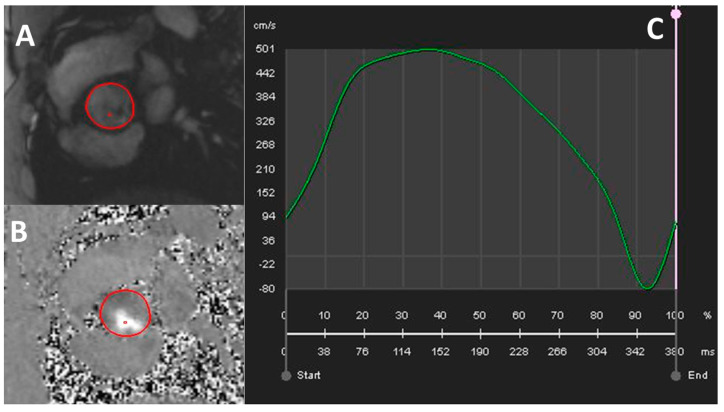
Typically, a phase-contrast velocity mapping acquisition produces two cine images, a magnitude (**A**) and a phase (**B**) cine. The magnitude image resembles a white blood gradient echo sequence in which the anatomic structures in the selected slice (perpendicular to the aortic orifice, generally at the level of the sinotubular junction; red circle) are displayed. The phase image is presented as a grayscale picture. In this through-plane acquisition, cranial systolic forward flow in the aortic root is displayed as white (it may be represented as black depending on how the sequence is programmed). Ambiguous background noise from the lungs appears as random black and white pixels in the phase image. (**C**) Time velocity curve generated by the CMR phase-contrast velocity mapping in a region of interest (in this case the sinotubular junction; red circle). VMax reaches 5 m/s which is consistent with severe AS.

CMR allows both anatomical and functional evaluation of AV (Figure 6). LV tissue characterization and quantification of aorta diameter is also possible by CMR imaging [44]. CMR is the reference technique for ventricular volumes, LVEF, segmental wall thicknesses, mass, and LV remodeling pattern assessment; therefore, it can be a good alternative to TTE in selected patients (i.e., those with a limited acoustic window). LV tissue characterization can help stratify patients according to their myocardial response to AS in terms of fibrosis and morphological and functional cardiac adaptation [45]. The principal applications of CMR are listed below:

- Planimetry. Cine sequences, selecting a perpendicular plane to the AV orifice, can be used to obtain a direct planimetry of the AVA. Such a measurement has been shown to be reproducible and correlates well with the AVA obtained by planimetry with 2D TEE [46] and with AVA estimated by catheterization [47]. Like MDCT, CMR planimetry slightly overestimates AVA compared to TTE [48]. Using Hakki’s formula, which is a simplification of Gorlin formula [49], AVA is mildly underestimated [48,50] compared to catheterization. Despite intra- and interobserver reproducibility of AV planimetry by CMR is excellent [48,50], its application is limited in certain scenarios such as the presence of a non-planar orifice, a highly calcified AV or arrhythmias (i.e., AF) [45].
- Functional AV assessment. In contrast to MDCT, functional assessment with CMR is feasible. Valenti et al. [51] suggested that the transaortic gradient can be indirectly calculated by using the simplified Gorlin equation (cardiac output/AVA). Such an estimation, in which the components of the formula are obtained using CMR cine imaging, is reproducible and has a good correlation with LV mass [51]. Phase contrast (PC) sequences are, nowadays, the most frequently employed for AV functional evaluation; VMax and, by using the simplified Bernoulli equation, maximum gradient are easily obtained with PC. These estimates have shown a high correlation with those obtained using Doppler technique and with invasive measurements [52,53]. Eccentric blood flow represents a challenge for PC-CMR, as a non-perpendicular plane alignment may underestimate AV velocity and, therefore, downgrade the stenosis [45]. Troger et al. [54] studied 55 patients with moderate or severe AS defined by cardiac catheterization (CC). All patients underwent CMR, TTE, and catheterization. AVA via PC-CMR was calculated as (flow − volume/VMax) during systole, and image planes parallel to the aortic leaflet attachment plane (LAP) were evaluated via PC-CMR between 22 mm below and 24 mm above LAP. AVA assessed in image planes 0–10 mm above LAP differed significantly from invasive measurements. Conversely, AVA values obtained 10–20 mm above LAP showed good agreement with invasive measurements; a plane 15 mm above LAP resulted in the lowest bias [54].

- Left ventricular tissue characterization. Applying gadolinium contrast and T1 mapping sequences, CMR can quantify areas of focal replacement fibrosis (they usually have a nonischemic pattern with a midwall location) and diffuse interstitial fibrosis, both resulting from myocyte apoptosis in the advanced stages of the disease. These types of fibrosis are correlated with the development of heart failure and unfavorable prognosis in patients with AS [55,56,57,58,59,60]. The ongoing EVOLVED trial will determine whether early aortic valve replacement in asymptomatic patients with severe aortic stenosis and midwall late gadolinium enhancement improves outcomes [61]. CMR is a great tool for the screening of amyloidosis in AS which occurs in one of eight patients evaluated for TAVR and carries an adverse prognosis [62].

The main limitations of CMR are its low availability in non-tertiary hospitals, contraindication for gadolinium contrast in patients with advanced kidney disease, and the inability to perform the test on claustrophobic patients and on those with a noncompatible CMR device. CMR currently has a limited role in cases of LF states [48]; recent position papers and guidelines do not include this technique in the assessment of patients in such scenarios.

## 3. Use of Multimodality Cardiac Imaging in Challenging Cases

In many cases, a standard TTE testing is enough to determine the AS grade, as the main echo parameters (AVA, VMax, and MG) agree. Sonographers must examine all TTE windows to obtain the highest VMax and MG with CWD because up to 20% of the maximal values are reached with non-apical views (NAV) [17]. Our group has recently published evidence that CWD assessment from NAV adds value over apical view evaluation; a total of 34% of patients are reclassified upwards and 19% are regraded from non-severe to severe AS when adding NAV to the TTE scan protocol. In accordance with prior papers, our data support the right parasternal view (RPV) as the NAV of choice due to its superior performance [63].

Minners et al. [64], in a cohort of more than 2000 patients with AS and preserved left ventricular ejection fraction (LVEF), analyzed the consistency among AVA, VMax, and MG. Correspondence between VMax and MG was very high (>90%). However, VMax or MG and estimated AVA demonstrated only 70% and 75% AS grading consistency, respectively. Investigators found that low-flow states contributed to discrepancies; nevertheless, 48% of inconsistently graded patients had a normal SVi > 35 mL/m^2^ [64]. It was found that an AVA of 1.0 cm^2^ correlated with an MG of 21 mmHg and a VMax of 3.3 m/s. Conversely, an MG of 40 mmHg was found to correspond to an AVA of 0.75 cm^2^, while a VMax of 4.0 m/s was found to correspond to an AVA of 0.82 cm^2^. These results questioned the value of the current cut-off points for the classification of AS. In fact, severity of AS by AVA was closer to 0.8 cm^2^ than to 1.0 cm^2^ [64].

**Figure 7:**
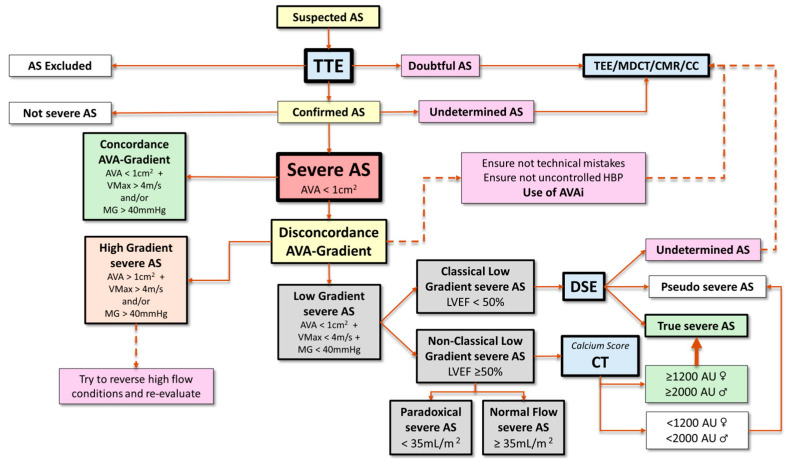
Central Illustration. When AS is suspected, an optimal TTE must be the first imaging test to be performed, including apical and non-apical views to evaluate flow velocities and pressure gradients. If AS is confirmed, grading parameters should be evaluated, as well as their concordance. As a result, three groups are established: concordant severe AS (green box), discordant high-gradient severe AS (orange box), and discordant low gradient (LG) severe AS (grey box). Discordant severe AS need further assessment. In the case of discordant high-gradient AS, it is mandatory to exclude high-flow conditions and, if those are present, re-evaluate AS under normal flow if such conditions can be amended. Discordant LG AS can also be subdivided into three subgroups. Classical LF–LG severe AS benefit from DSE to differentiate true severe from pseudosevere AS. Paradoxical and NF–LG AS should be evaluated with MDCT (calcium score). Finally, when undetermined results are obtained, further assessment using alternative imaging tests (i.e., TEE, CMR) can be helpful to reach a final AS grading. AS: aortic stenosis. AU: Agatston units. AVA: aortic valve area. AVAi: indexed AVA. CC: cardiac catheterization. CMR: cardiac magnetic resonance. CT: computed tomography. DSE: dobutamine stress echocardiography. HBP: high blood pressure. MDCT: multidetector computed tomography. MG: mean gradient. TEE: transesophageal echocardiography. TTE: transthoracic echocardiography. VMax: transvalvular flow maximal velocity.

Bearing in mind the previous considerations, we will now focus on key aspects that need to be taken into account for the resolution of cases of AS and discordant classification through the use of MCI (see Figure 7—Central Illustration).

### 3.1. Inadequate Doppler Alignment and Lack of Acoustic Window

If proper CDW alignment with a transvalvular flow cannot be achieved even after interrogating NAV with a 2D TTE probe, a blind transducer (PEDOF probe) may be helpful. Due to its small size, the PEDOF probe allows for better positioning and angulation than 2D transducers, facilitating parallel alignment of the US beam with the transaortic jet [65]. Training and experience are required to handle a blind transducer, as there is no anatomical reference for orientation (the probe lacks a 2D image). For example, it is not uncommon to misinterpret the aortic ejection flow with a jet of mitral regurgitation (MR) or tricuspid regurgitation (TR). The duration (width) of the spectral curves must be taken into consideration; MR and TR signals, due to the existence of higher diastolic pressure in the AV than in the atria, appear during isovolumetric contraction, that is, with the beginning of the QRS of the electrocardiogram (ECG), unlike the aortic spectrogram, which appears later.

In patients without an acoustic window, TTE is useless and other imaging techniques such as TEE, MDCT, or CMR are mandatory.

### 3.2. Discordant High Gradient Severe Aortic Stenosis (AVA ≥ 1 cm^2^ with VMax ≥ 4 m/s and/or MG ≥ 40 mmHg)

These cases are usually explained by the presence of a high-output condition (e.g., due to anemia, fever, thyrotoxicosis, an arteriovenous shunt for hemodialysis, etc.). If so, echocardiographic staging should be repeated if the cause is identified, reversible, and under control. In situations where the etiology of the high-output state is not reversible, AS should be classified as severe, placing the VMax–MG criterion ahead of AVA, since several studies have shown that the prognosis of these patients is similar to that of patients with high gradient AS and AVA < 1 cm^2^ [66].

### 3.3. Discordant Low Gradient Severe aortic Stenosis (AVA < 1 cm^2^ with VMax < 4 m/s and MG < 40 mmHg)

This entity can be divided into three different phenotypes according to LVEF and Svi (see Table 2). The first step in these cases is to verify that the TTE (scanning and post-processing) has been performed correctly and there are no errors. As pointed out, special care must be taken when measuring the diameter of the LVOT. After reviewing the TTE examination and correcting possible mistakes, if severe discordant and low-gradient AS persists, different approaches should be taken depending on the subtype.

#### 3.3.1. Classical Low-Flow Low-Gradient Severe Aortic Stenosis

**Figure 8:**
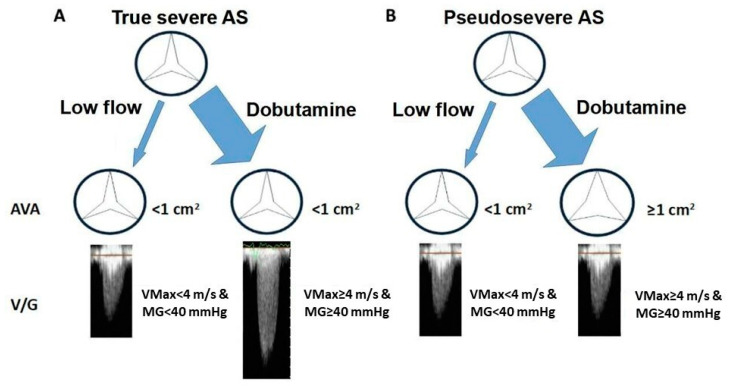
Response to dobutamine in patients with classic low-flow low-gradient severe AS. When dobutamine significantly rises stroke volume (>20% compared to baseline) two different responses can be obtained: (**A**). gradient increases while maintaining a reduced AVA (<1 cm^2^); AS is severe, (**B**). a relevant increment in gradient is not observed and AVA ≥ 1 cm^2^; AS is pseudosevere. AS: aortic stenosis. AVA: aortic valve area. MG: mean gradient. VMax: transvalvular flow maximal velocity.

The gradient across the VA is pseudonormalized and underestimates the degree of AS due to systolic ventricular dysfunction, which may be secondary to the AS itself or to the presence of a concomitant cause such as an extensive coronary artery disease or a dilated cardiomyopathy. This variant accounts for 5–10% of the patient population with AS and is the one with the worst prognosis, with a mortality between 40% and 60% after two years without treatment. As mentioned above, when discussing the CEq, it is important to consider that patients with left ventricular systolic dysfunction may sometimes have a reduced AVA due to the inability of the left ventricle to overcome the inertia to maximal AV opening (pseudosevere AS). Distinguishing the two entities, true severe AS and pseudosevere AS, is important because valve intervention is beneficial in the former but not in the latter, in which treatment should be focused on ventricular dysfunction. The test of choice for this purpose is dobutamine stress echocardiography (DSE). In patients with severe AS and contractile reserve (defined as an increase in SV > 20% compared to baseline), dobutamine infusion increases VMax and GM, reaching the threshold of severe AS without significant fluctuations being observed in AVA (increase < 0.3 cm^2^), which remains < 1 cm^2^. However, in pseudosevere AS, SV improvement is accompanied by an increase in the AV opening, resulting in an AVA > 1 cm^2^ without relevant gradient variations (see Figure 8). In some cases, especially in patients without contractile reserve, a change in AVA and gradients may not be apparent, and, therefore, it may not be possible to determine whether the AS is truly severe or not. In these situations, calculating a projected AVA at a normal flow rate (i.e., 250 mL/s) can be useful [67,68,69]. Lack of contractile reserve is associated with poor outcomes and high surgical mortality [70]. Expert consensus recommends the addition of another technique to confirm the severity of AS (i.e., MDCT calcium score) and consider intervention (TAVR) in symptomatic patients [4].

#### 3.3.2. Non-Classical Low-Gradient Aortic Stenosis: Paradoxical and Normal Flow

**Figure 9:**
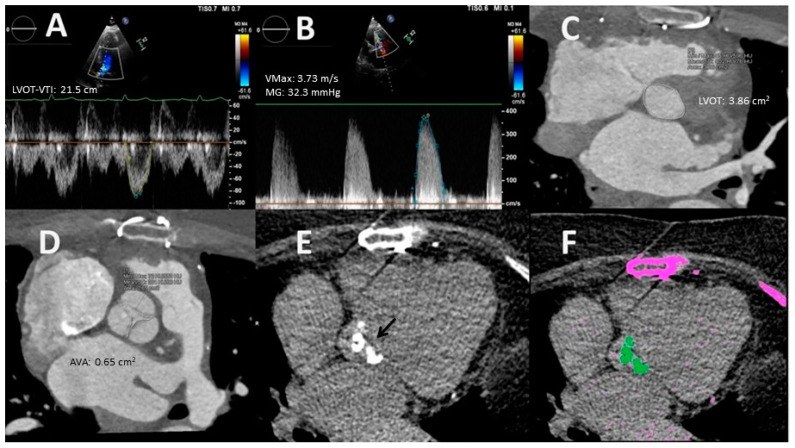
Example of a challenging AS scenario. This 80-year-old woman presented with symptomatic AS, which was initially classified as NF–LG severe AS based on TTE results. (**A**) A5C view: LVOT–VTI assessment. (**B**) The best transvalvular flow alignment was obtained from RPV, but VMax was <4 m/s (3.73 m/s). (**C**) LVOT planimetry by MDTC (3.86 cm^2^). AVA estimated by the hybrid method was 0.92 cm^2^. (**D**) AV direct planimetry by MDTC (AVA = 0.65 cm^2^). (**E**) Non-contrast CT image showing calcification of the trileaflet aortic valve (black arrow). (**F**) AV calcium score quantification; AV calcium is highlighted in green (1332 AU). AS was finally graded as severe. TAVR was performed without complications. The patient remained asymptomatic at two-years follow up. AV = aortic valve; AVA = aortic valve are; AS = aortic stenosis; AU = Agatston units; A5C = apical-5-chambers; CT = computes tomography; LVOT = left ventricular outflow tract; LVOT-VTI = left ventricular outflow tract velocity time integral; MDCT = multidetector cardiac tomography; NF–LG = normal-flow low-gradient; RPV = right parasternal view; TAVR = transcatheter aortic valve replacement; TTE = transthoracic echocardiography; VMax = transvalvular flow maximal velocity.

NF–LG severe AS is a variant that has classically been considered a pseudosevere AS in relation to TTE measurement errors. In fact, the latest clinical practice guidelines on the management of valvular diseases are positioned in this sense, indicating that the probability of true severe AS in this setting is low, and recommend management of these cases as moderate AS [71]. Two aspects draw attention to NF–LG severe AS: (a) its high prevalence (15–40% of AS), a finding that is difficult to attribute solely to calculation faults, and (b) the fact that phenomena such as a low aortic compliance and high blood pressure may lead to a substantial reduction in AV gradient and justify the presence of true severe AS with this hemodynamic profile [72]. These circumstances suggest that this type of AS represents a particular disease which requires a thorough evaluation rather than a misclassified mild/moderate AS as it has been suggested until now (Table 3). Generally, patients falling within this category are associated with lower mortality rates than those associated with other types of severe AS [73]. Reported results on valve intervention are controversial; while some studies have not found significant differences in survival compared to medical treatment, others, including a meta-analysis, have shown benefits [74]. Such disparate findings are in favor of a very heterogeneous population with a percentage of true severe AS that can reach up to 40–50% [72] that should be additionally studied with further imaging test (see Figure 9) [39].

A high proportion of patients with AS show preserved LVEF and low-flow state (≈25–35%). In these cases, the SV reduction is related to an accentuated concentric remodeling that generates a small left ventricular cavity. Alterations in diastolic function and decrease in myocardial deformity (longitudinal strain), a finding that indicates an incipient alteration in systolic function [75], are common. This subgroup of AS has many pathophysiological similarities with heart failure with preserved LVEF and, from a clinical point of view, both are associated with a similar patient profile: advanced age, female sex, and hypertension. Mortality observed in patients with severe paradoxical AS may be even higher than that associated with patients with a high gradient, with a clear benefit from valve intervention [76]. Differential diagnosis with pseudosevere AS, which can represent 30–40% of cases, is required. Nowadays, the degree of AV calcification using MDCT is the parameter of choice in this regard (see Figure 7).

**Table 3:** Integrated approach to the assessment of challenging AS. Findings suggestive of severe AS [4,14].

Clinical findings	Crescendo–decrescendo systolic murmur auscultated at the right upper sternal border (may radiate to the carotid arteries) with reduced intensity of the second heart sound [77] Typical symptoms (dyspnea, angina, or syncope) without other explanations Elderly patients (>70 years)
Qualitative imaging data	LVH (additional history of hypertension to be considered) Reduced LV longitudinal function (global longitudinal strain) without other explanations
Semiquantitative imaging data	CW waveform: slow acceleration with a late peak Extensive calcification Increased cusps thickness with restrictive motion
Quantitative imaging data	MG > 35 mmHg AVA < 0.8 cm^2^ High calcium score by MDCT

### 3.4. Cardiac Rhythm Abnormalities

For VMax, MG, and VTI calculations in patients with sinus rhythm, it is recommended to average three beats [4,14]. Regarding AF, the number of consecutive cardiac cycles to be assessed should be increased to at least five. Using such a number of beats implies reducing the sweep speed, a factor that increases the risk of mismeasurements. To solve this issue, new approaches such as the employment of single but matched R–R intervals are proposed [78].

### 3.5. Combined Heart Valve Disease

AS does not usually appear as an isolated heart valve disease. Mitral valve disease (stenosis or regurgitation) or aortic regurgitation (AR) can often be present.

#### 3.5.1. Aortic Regurgitation

Common etiological factors justify the coexistence of AS and AR. However, regurgitation is usually mild and has little impact on the assessment of AS. When relevant AR develops, the transaortic flow rate increases and VMax and MG rise, but they do not reflect the effective SV [76]. Current recommendations support, however, VMax in mixed AV disease as a main parameter for clinical decisions making [4,14] because it reflects both stenosis and regurgitant severity and has been shown to predict event-free survival [79].

#### 3.5.2. Mitral Regurgitation

A combination of AS and MR is common in older people [80]. If both valvular diseases are present, we should carefully examine each individual lesion. In MR, it is important to distinguish between primary and secondary aetiology. Regarding AS, in cases of significant MR, the transaortic flow rate may be reduced, so VMax and MG may underestimate the severity of the stenosis. In this context, AVA is recommended over flow parameters as well as MCI. Zilberszac et al. [81] examined 89 symptomatic patients with severe AS and at least moderate MR (mostly secondary). Addition of surgical treatment if severe concomitant MR was present to AV replacement improved survival in an unadjusted population but did not reach statistical significance after propensity matching. A meta-analysis has shown that concurrent moderate-to-severe MR is associated with increased early and late mortality after TAVR. However, it is noteworthy that half of the investigated patients developed significant improvement in MR severity after the percutaneous procedure [82]. It would be important to determine which cases presenting both valve lesions may benefit from a dual intervention; data remain scarce and further investigations are required [83].

#### 3.5.3. Mitral Stenosis

Concomitant mitral stenosis (MS) and AS are strongly associated with rheumatic valve disease. MS is a well-recognized aetiology of LF–LG AS. AVA better predicts severe AS in this circumstance [84], and planimetry with TEE may be useful as it can estimate both AVA and mitral valve area (MVA) within the same procedure. Kato et al. [85] retrospectively analyzed patients who had undergone AV replacement (surgery of TAVR) for AS with a coexistent MS. MVA improved after AVR in nearly half of the patients (“pseudosevere MS”). Conversely, individuals with a true MS showed higher mortality [85]. In contrast, the investigation led by Yamashita et al. [86] concluded that MS was not a mortality risk factor in patients with severe AS who had undergone TAVR. These conflicting results highlight the need for additional studies.

## 4. Current Gaps and Future Directions

- Non-classical low-gradient severe AS: information about its prognosis and treatment benefits is limited and controversial. MCI could play a key role in unmasking true severe AS cases.
- Low-flow low-gradient AS without contractile reserve: evidence on management is very scarce. More studies aimed at better understanding this entity are required.
- Combined heart valve disease: this clinical scenario has not been extensively analyzed. Further investigations are needed to increase our knowledge about these pathologies and clarify the best therapeutical options.

## 5. Conclusions

In many cases, severe aortic stenosis is not a straightforward diagnosis. In all these challenging scenarios, multimodality cardiac imaging can lead to a higher precision grading and a better decision-making process.

## Figures and Tables

**Table 2:** Types of severe LG AS. Source: own elaboration.

LG AS: AVA < 1 cm^2^, VMax < 4 m/s and MG < 40 mmHg		
Type	SVi	LVEF
Classical LF–LG	<35 mL/m^2^	Reduced (<50%)
Paradoxical LF-LG	<35 mL/m^2^	Preserved (≥50%)
NF–LG	>35 mL/m^2^	Preserved (≥50%)

## Data Availability

Not applicable.
